# Patient Satisfaction With Breast Cancer Care Delivery at the National Cancer Institute of Sri Lanka: Does Language Play a Role?

**DOI:** 10.1200/GO.22.00366

**Published:** 2023-02-23

**Authors:** Susan J. Thanabalasingam, Sarith S. Ranawaka, Sathika S.C. Gunarathna, Bala Yathev, Christopher M. Booth, Sanjeewa Seneviratne, Sanjeeva Gunasekera, Don Thiwanka Wijeratne

**Affiliations:** ^1^ Kingston Health Sciences Centre, Kingston, ON, Canada; ^2^ Department of Medicine, Queen's University, Kingston, ON, Canada; ^3^ Faculty of Medicine, University of Colombo, Colombo, Sri Lanka; ^4^ Department of Oncology, Queen's University, Kingston, ON, Canada; ^5^ National Cancer Institute, Colombo, Sri Lanka

## Abstract

This study sought to examine whether there was an association between language barriers and patient satisfaction with breast cancer care in Sri Lanka.

**METHODS:**

A telephone-based survey was conducted in the three official languages (Sinhala, Tamil, or English) among adult women (older than 18 years) who had been treated for breast cancer within 6-12 months of diagnosis at the National Cancer Institute of Sri Lanka. The European Organisation for Research and Treatment of Cancer Satisfaction with Cancer Care core questionnaire was adapted to assess three main domains (physicians, allied health care professionals, and the organization). All scores were linearly transformed to a 0-100 scale, and subscores for domains were summarized using means and standard deviations. These were also calculated for the Sinhalese and Tamil groups and compared.

**RESULTS:**

The study included 72 participants (32 ethnically Tamil and 40 Sinhalese, with 100% concordance with preferred language). The most commonly reported best aspect of care (n = 25) involved affective behaviors of the physicians and nurses. Ease of access to the hospital performed poorest overall, with a mean satisfaction score of 54 (30.5). Clinic-related concerns were highlighted as the worst aspect of the care (n = 10), including long waiting times during clinic visits. Sixty-three percent of Tamil patients reported receiving none of their care in Tamil and 18% reported experiencing language barriers during their care. Tamil patients were less satisfied overall and reported lower satisfaction with care coordination (*P* = .005) and higher financial burden (*P* = 0.014).

**CONCLUSION:**

Ethnically Tamil patients were significantly less satisfied than their Sinhalese counterparts and experienced more language barriers, suggesting there is a need to improve access to language-concordant care in Sri Lanka.

## INTRODUCTION

Breast cancer is the most common malignancy among females in Sri Lanka.^1,2^ The incidence of breast cancer is rising, with a gradual increase of approximately 4% per year observed between 2001 and 2010.^3^ Patient satisfaction with breast cancer care remains understudied in Sri Lanka, despite having a publicly funded health care system with universal access.^4^

**CONTEXT**

**Key Objective**
Do language barriers influence patient satisfaction with breast cancer care provision in Sri Lanka?
**Knowledge Generated**
Tamil patients, who are an ethnic minority in Sri Lanka, experienced more language barriers during their breast cancer care. Although satisfaction levels were generally high overall, Tamil-speaking participants did report significantly lower patient satisfaction with overall care compared with Sinhala-speaking patients.
**Relevance**
Language barriers may play an important role in patient satisfaction with cancer care, suggesting the need for steps toward better access to language-concordant care.

Patient satisfaction is a valid, indispensable measure of quality of care.^5^ It is an important quality indicator that may not necessarily align with other indicators of health care access and clinical outcomes. Several studies have reported there may be a relationship between patient satisfaction and perceived health care–related quality of life in the oncology setting.^6-8^ Assessment of patient satisfaction is becoming increasingly important in oncology to optimize patient-centered care. Tools to evaluate patient experience to ascertain satisfaction are generally implemented with multidimensional surveys or questionnaires—these may help inform strategies to improve care delivery.^9^

The provision of patient-centered care is fundamentally tied to linguistically and culturally competent communication—when a health care provider and a patient do not share a common language or culture, communication difficulties that arise can have adverse consequences.^10^ A demonstrated association between patient dissatisfaction with care and language barriers highlighted an unmet need among patients with cancer for linguistically sensitive care provision.^11^ Health inequalities because of language barriers are mitigated when care providers are fluent in the patient's preferred language; hence, language-concordant care has been shown to improve outcomes.^12^

Sri Lanka is a multiethnic society including the Sinhalese majority, the Tamils, the Moors (a predominantly Muslim community), and the Burghers, who are of mixed European descent. Sinhala, Tamil, and English are all official languages in Sri Lanka and are variably spoken by these ethnic groups. Importantly, English is not commonly used in the publicly funded health care setting in Sri Lanka. Approximately 74.9% of the population identifies as ethnically Sinhalese and 87% of the population are Sinhala-language–speaking, compared with 11.2% and 28.5% identifying with Tamil ethnicity and Tamil language, respectively.^13^ Whether linguistically and culturally concordant care is accessible to patients, and whether their satisfaction with breast cancer care delivery is influenced by language proficiency, remain unknown. To these ends, we embarked on a survey-based study to further evaluate satisfaction with breast cancer care delivery among patients at a tertiary care center in Sri Lanka, comparing experiences between Tamil and Sinhalese patients.

## METHODS

### Study Population and Setting

We performed a telephone-based survey among adult women who had been treated for breast cancer at the National Cancer Institute of Sri Lanka (NCISL). Those who were within 6-12 months from the time of their diagnosis were considered eligible for inclusion to account for completion of most of their care. Cancer treatment in Sri Lanka is centralized at the NCISL, with more than 40% of patients with cancer rotating through this center, making it a good location to have a representative sample of patients. Cohorts of participants were randomly generated using a computer-based sample selection from a prospective cohort created by our group that includes more than 6,000 patients with breast cancer to date. All participants had consented to be contacted for research purposes at the time their data were registered in the Cancer Sri Lanka (CSL) Database. Verbal consent to participate in the telephone interview was obtained before proceeding with the call. The study was approved by the ethics review committee of the Faculty of Medicine, University of Colombo, Sri Lanka.

### Data Collection

The CSL database contains comprehensive data for all patients who received breast cancer care at the NCISL.^14^ These data were securely matched to study participants and collated for analysis. The European Organisation for Research and Treatment of Cancer (EORTC) *Satisfaction with Cancer Care core questionnaire* (PATSAT-C33) was adapted for our use to broadly assess patient satisfaction. The original PATSAT-C33 questionnaire is currently undergoing phase IV large-scale cross-cultural psychometric assessment.^15^ Additionally, the equivalent inpatient EORTC questionnaire was previously evaluated in the Sinhala language and found to be a valid measure of cancer care satisfaction across heterogeneous cancer sites.^16^ Author SJT developed a Tamil-language translation of the original English-language draft of the adapted survey. It was subsequently retranslated to English by an independent native Tamil speaker to ensure interlingual validity. A Sinhala-language translation was similarly developed by authors SSR and SG.

In our adapted version of the EORTC-PATSAT C33 questionnaire, we explored the same three main domains as the original survey, which addressed physicians, allied health care professionals, and the service and care organization. Physicians were evaluated on their technical skills (five items), information exchange (six items) and affective behaviors (five items), and their ability to prognosticate (three items). Allied health team members were assessed on their information provision and responsiveness (three items), and their affective behaviors (four items). The hospital service organization questions encompassed coordination (three items), interaction with the health care team (seven items), and five other single items. We additionally included a section to gauge the financial burden of illness (three items), along with a single-item question addressing perceived level of safety while receiving care. These domains were assessed using closed-ended questions with each individual item rated on a 5-point Likert scale (1 = very poor, 2 = poor, 3 = average, 4 = good, and 5 = excellent; Data Supplement). There were four open-ended questions inviting participants to further elaborate on their experience. Additional questions to ascertain language proficiency, preference, and language of care delivery were included as the CSL Database stores information on ethnicity, which does not necessarily directly correlate with language proficiency in Sinhala and Tamil.

The questionnaire was administered via telephone by authors S.S.R., S.S.C.G., and B.Y. Participants were given the option to have the questionnaire administered in Sinhala, Tamil, or English, as per their individual preference. Responses were recorded for the purposes of data collection in the English language.

### Statistical Analysis

For baseline demographic data, continuous variables are represented as means with standard deviations, and categorical variables are represented as numbers and percentages. All scale and single-item scores were linearly transformed to a 0-100 scale, with a higher score indicating a higher degree of satisfaction. The scoring system suggested by the EORTC was adapted for use in our analysis.^17^ Subscores were descriptively summarized using means and standard deviations. Responses for each score domain were examined within each ethnic cohort and compared using a Student's *t* test for independent samples. A *P* value of < .05 was considered statistically significant.

## RESULTS

This study included 72 participants, of whom 32 were ethnically Tamil and 40 Sinhalese. All patients who were approached participated in this study. There were no respondents in English. Baseline demographic characteristics (Table 1) are summarized for the entire study population, and stratified by ethnicity. Median age at the time of diagnosis was 61 (31-81) years and was 64 (33-84) years at the time of survey administration. Overall, the Sinhalese and Tamil subgroups had a similar demographic profile.

**TABLE 1:**
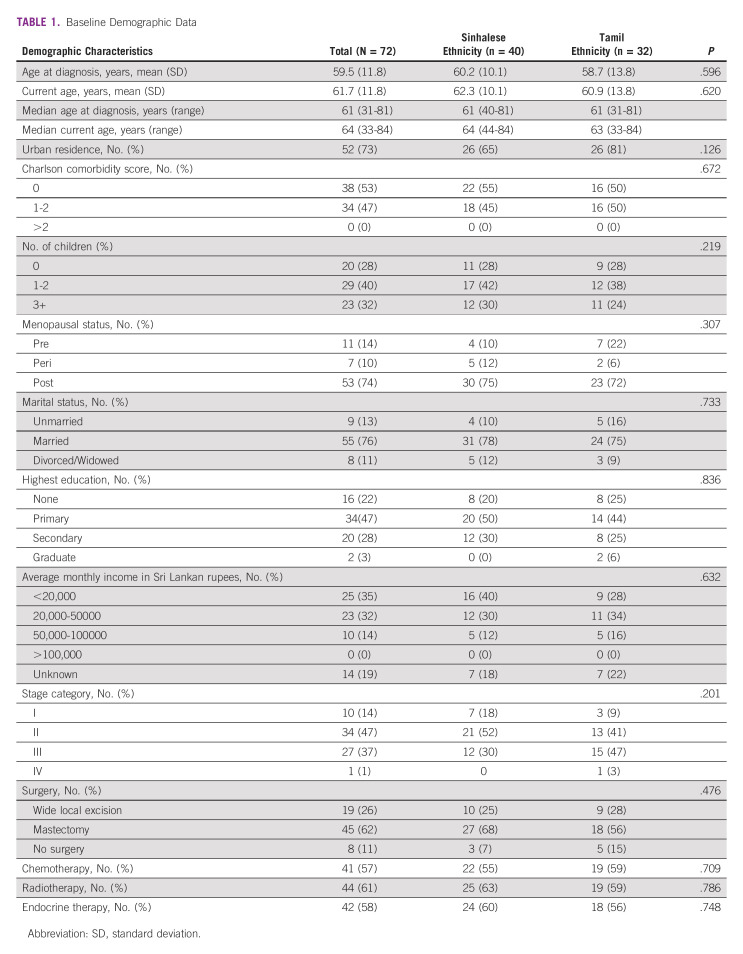
Baseline Demographic Data

Socioeconomic status was gauged using self-disclosed average family monthly income and highest education level attained by the patient. These were similar overall between the two groups apart from more patients with average monthly income <20,000 Sri Lankan rupees (SLR) in the Sinhalese group. Most participants (67%) had monthly income <50,000 SLR. The highest level of education for much of the cohort was primary (47%), with similar proportions having done no schooling at all (22%) and having completed secondary education (28%).

With respect to breast cancer disease status at the time of diagnosis for the overall cohort, most participants had stage II (47%) or stage III (37%) disease at the time of diagnosis. Most participants were treated with mastectomy (62%), chemotherapy (57%), radiation (61%), and endocrine therapy (58%) without substantial differences in treatment modalities between the two ethnic groups.

### Language proficiency and preference

In this study population, there was complete concordance between participants' ethnicity and primary language—all patients with Sinhalese ethnicity in the database identified Sinhala as their mother tongue and preferred language of communication, and all ethnically Tamil patients identified Tamil as their mother tongue. Overall, Tamil patients reported significantly lower Sinhala proficiency compared with Sinhalese patients (*P* < .001). Fifteen Tamil participants (21%) reported having a caretaker fluent in Sinhala helping them communicate with health care providers during appointments. Most patients had < 50% of their care delivered in the Tamil language (n = 65; 90.2%), with 58 respondents (81%) reporting total care provision in Sinhala. Of the 32 Tamil patients, 20 (63%) reported receiving none of their care in Tamil. The average conversational Sinhala fluency for these 20 participants corresponded to a Likert rating of 2.65 out of five (between poor and average). Seven (10%) patients reported >50% care provision in Tamil, with only one patient reporting total care provision in Tamil. Thirteen (18%) respondents reported experienced a language barrier during their care provision, with three patients classifying this barrier as severe. All 13 of these respondents identified Tamil as their primary language.

#### Adapted EORTC-PATSAT-C33 scores.

Table 2 summarizes mean scores for the entire cohort and stratified by ethnicity. Patient satisfaction was good overall, with no domain or single-item scores <50, except for Sinhala proficiency among Tamil patients. Patients were generally very satisfied with their overall care with a mean standard deviation (SD) of 83 (19). Satisfaction was relatively higher in the allied health domains (information exchange and affective behaviors) compared with the physician domains (technical skills, information exchange, and ability to prognosticate) apart from physicians' affective behavior (76 [19]), which performed similarly to allied health affective behaviors (75 [21]). Ease of access to the hospital performed poorest overall, with a mean satisfaction score of 54 (31). Patients were most satisfied (88 [17]) with respect to the level of safety they felt while receiving care.

**TABLE 2:**
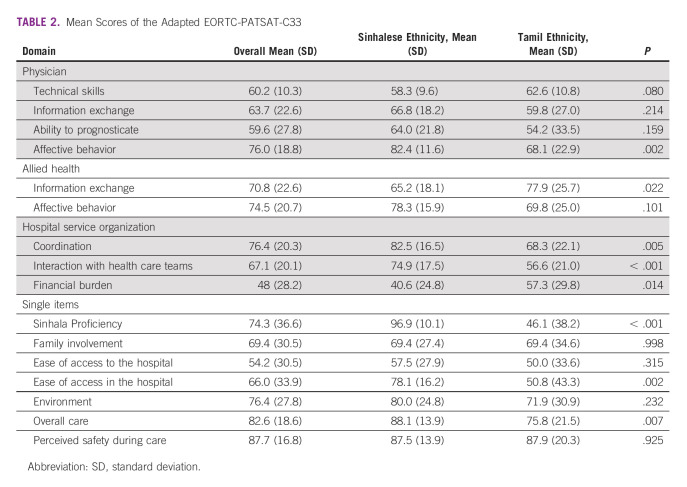
Mean Scores of the Adapted EORTC-PATSAT-C33

Tamil and Sinhalese respondents reported similar levels of satisfaction regarding their physicians' technical skills, information exchange, and ability to prognosticate. There was a significant between-group difference for satisfaction with physicians' affective behaviors. Tamil participants were significantly less satisfied, with a mean score of 68 (23), than their Sinhalese counterparts (82 [12]) in this domain (*P* = .002). There was no significant between-group difference in the allied health affective behavior domain, but Sinhalese respondents were less satisfied with allied health information exchange. There were significant differences in the coordination, interaction with health care teams, and financial burden domains. Tamil patients reported lower satisfaction with care coordination (*P* = .005) and health care team interactions (*P* < .001), and higher financial burden (*P* = 0.014).

For single-item scores, there were no significant differences in mean satisfaction between the two subgroups regarding family involvement in care, ease of access to the hospital, safety, and the physical hospital environment. Sinhalese patients were significantly more satisfied with their overall care experience (*P* = .007) and navigating the hospital (*P* = .002) than their Tamil counterparts.

#### Open-ended responses.

**FIG 1:**
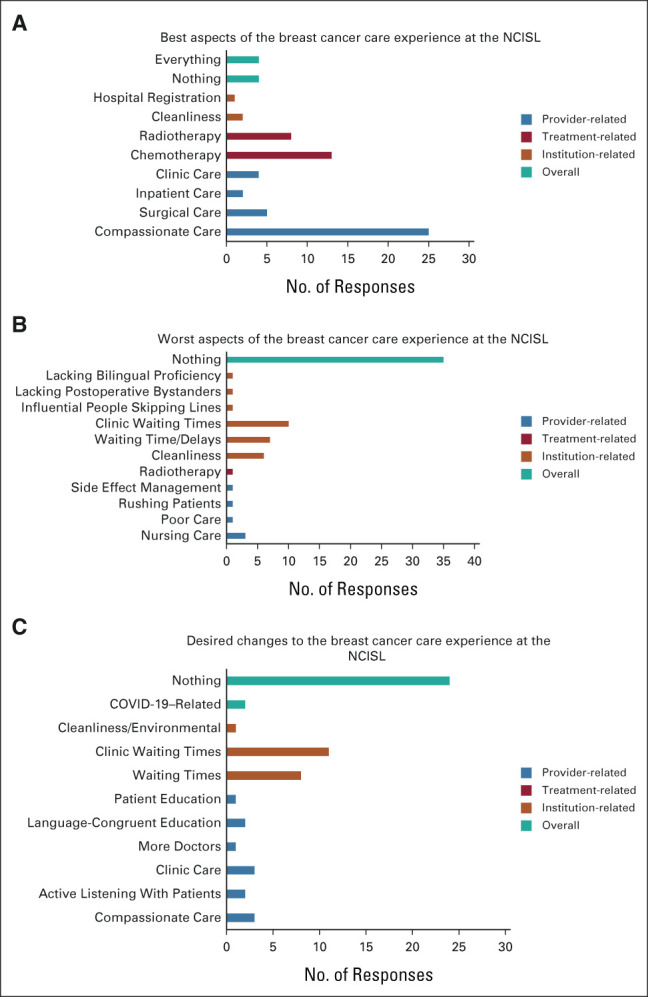
Summary of open-ended survey responses on patient satisfaction. (A) Best aspects of the breast cancer care experience at the NCIS. (B) Best aspects of the breast cancer care experience at the NCISL. (C) Desired changes to the breast cancer care experience at the NCISL. NCISL, National Cancer Institute of Sri Lanka.

Responses to the three open-ended questions included in the survey are visually summarized in Figure 1.

##### Best aspect of the care.

A minority (n = 4) of participants identified nothing, while four other respondents reported everything as the best aspect of their care experience. The most common response (n = 25) centered on the affective behaviors of the physicians and nurses, with specific comments on the kindness, patience, and compassion they demonstrated. Chemotherapy (n = 13) and radiotherapy (n = 8) were also identified by several participants as the best aspects of their care experience. A minority also highlighted intraoperative (n = 3) and postoperative care (n = 2). Hospital cleanliness (n = 2), clinic-related care (n = 4), and inpatient care provided during hospital admissions (n = 2) were also emphasized. Care after the occurrence of complications, the registration center, and early hospital discharge were each reported by one patient as the best aspects of their care.

##### Worst aspect of the care.

Thirty-five (49%) respondents reported northing as the worst aspect of their care experience. Several participants (n = 10) highlighted clinic-related issues. Concerns included long waiting times during clinic visits, overly crowded clinics, and lacking social distancing in clinic spaces. Waiting times and delays in care more generally were also highlighted by seven respondents. One participant specifically commented on the long waiting times for diagnostic imaging, and another felt there were care delays due to physicians leaving the ward. Environmental concerns (n = 6) involved unclean washroom spaces, with only one participant more generally commenting *smell and noise* without reference to a particular location. Nursing-related concerns were reported by a minority (n = 3) and centered on poor affective behaviors (“Some nurses disrespect the patients and family members” or “were very rude”) and delays in postoperative wound care. One respondent, whose primary language was Tamil, noted that the worst aspect of their care was the lack of people with bilingual proficiency and no preference for the handicapped. More generally, radiotherapy and poor side-effect management were also each reported by one respondent.

#### Desired changes to the care experience.

Twenty-four (33%) respondents reported wanting to change nothing about their care experience. The second most common response (n = 14) involved clinic-related concerns, namely lengthy wait times and overcrowding. Other clinic-related requests included interacting with consultants at clinic visits, physical examination of the patient by physicians at each clinic visit, and longer follow-up visits. Changes to the physical environment were also identified by several participants (n = 9), namely improving washroom cleanliness as well as upgrading hospital amenities. Eight respondents commented on wanting to change the lengthy waiting times and care delays. Six (8%) comments centered on the affective behaviors and technical skills of health care workers. The technical concerns included wanting more direct communication with patients, paying more attention to their symptoms, addressing concerns without saying they are usual/common, and more education around the investigations performed. The affective behavior changes included being friendlier with patients, reducing disrespect from nursing staff toward patients and family, and refraining from technical staff's impoliteness toward patients.

Two respondents highlighted changes focused on the language of care provision, specifically better explanations delivered in Tamil, and providing adequate knowledge on prevention and awareness in Tamil. There were three responses relating to the involvement of family members in care, namely providing care-related information to family members to reduce cognitive load on the patients themselves and providing accommodations for family.

### Missing Data

There were 27 missing responses to closed-ended questions, which represented 0.7% of all closed-ended survey responses. Two patients accounted for 17 of these missing responses. For seven missing responses, domain scores were calculated accounting for the single missing response.

## DISCUSSION

To our knowledge, this is one of the first studies to examine whether language of care delivery meaningfully affects patient satisfaction with oncology care in Sri Lanka. In this cohort of patients with breast cancer, overall satisfaction was significantly lower in the Tamil subgroup compared with the Sinhalese. However, the general satisfaction with care at the NCISL was quite high in our cohort. Our study also found that despite having access to universal health care, Sri Lankan patients with breast cancer experience moderate financial burden, with Tamil patients reporting higher burden than their Sinhalese counterparts. Compassionate care from health care providers was notably highlighted as one of the best aspects of the care experience at NCISL.

The significantly lower care satisfaction among Tamil patients in our cohort was consistent with our hypothesis that native Tamil speakers may be less satisfied than Sinhala speakers who are more likely receive language-concordant care. In this cohort, ethnicity correlated completely with the primary language of communication. Tamil patients at the NCISL appear to face significant language barriers during their oncology care provision, which we suspect was correlated with the lower overall satisfaction, and lower satisfaction with doctors' affective behaviors and interaction with health care teams.

Previous studies have demonstrated that ethnic and linguistic differences may lead to significant communication barriers in health care,^10^ specifically in oncology care.^18^ Patients with limited proficiency in the language of care provision are more vulnerable to experiencing inequities.^19^ As such, exploring patient satisfaction among patients of different language proficiencies has the potential to identify gaps in care and areas for improvement. Bregio et al^20^ recently explored this specifically in an oncology setting and found Spanish language-concordant interactions may facilitate more effective therapeutic relationships and biomedical counseling. Similar results to our own have been seen in the care of people with limited English proficiency, including Spanish-speaking^21,22^ and Asian^18,23^ patients in the United States. Increasing access to interpreters and better distribution of Tamil health care professionals would be important and potentially feasible next steps to begin mitigating these language barriers. Language-concordant patient navigators may also serve an important role in addressing these barriers to improve access and outcomes among minority groups.^24^

The overall financial burden of illness in our cohort was moderate despite Sri Lanka having a publicly funded universal health care system. This is consistent with a recent systematic review demonstrating that patients do incur substantial out-of-pocket expenses, including nonmedical ones such as travel or caregiving, in countries with universal free health care systems.^25^ This systematic review also found that this out-of-pocket cost burden was significantly higher in low- and middle-income countries compared with high-income countries, and among ethnic minorities, low-income patients, and rural dwellers.^25^ Congruently, we did find that Tamil patients in our cohort reported higher financial burden than their Sinhalese counterparts despite having higher self-reported average monthly income than the Sinhalese subgroup.

The high mean scores around affective behaviors of doctors and allied health professionals were reflected in the open-ended responses highlighting health care providers' care and compassion as the best aspect of their care experience, despite a significant workload noted among oncologists in Sri Lanka who on average see more than 30 patients per day.^26^ This high level of satisfaction with interpersonal interactions, particularly relating to nursing care, is consistent with a previous report from a nononcology Sri Lankan setting.^27^ There remain potential areas for improvement in this domain, as reported by a minority of respondents in this study (n = 3), who reported experiencing disrespect. Of note, all three participants who suggested health care providers' affective behaviors could be improved were all ethnically Tamil, although we cannot comment on whether there is any direct correlation between their experiences and ethnicity. Long wait times and environmental concerns, mostly related to restrooms, were also highlighted as key areas for improvement, which have been previously demonstrated in Sri Lanka.^27,28^

We identify several strengths of our study. To our knowledge, ours is one of the first studies to examine the impact of ethnicity and language on patient satisfaction in Sri Lanka, with good representation of Tamil and Sinhalese ethnicities in our real-world sample of patients. Another key strength of this study was the administration of an internationally validated survey that was adapted for our use. Our survey was also designed using neutrally phrased statements, which has been demonstrated to be important for limiting bias in patient satisfaction questionnaires.^29^ Bias was further limited through the administration of all surveys within 12 months of breast cancer diagnosis, thereby reducing recall bias. We also identify several limitations in this study. Although the survey was conducted in English, Tamil, or Sinhala in accordance with the participants' preferences, data were translated and transcribed in the English language by interviewers, which potentially introduced bias into the open-ended responses. Additionally, there were several missing responses with incomplete survey completion by a minority of participants. Another limitation was the relatively smaller sample sizes of the two ethnic groups, which prevented us from performing meaningful subgroup analyses within each group and limited the reproducibility of our results. The monocentric nature of our study also potentially limits the generalizability of our findings.

In conclusion, in this cohort of patients with breast cancer in Sri Lanka receiving care at the NCISL, although patient satisfaction was generally high overall, we did find ethnically Tamil patients were significantly less satisfied than their Sinhalese counterparts. Tamil participants did report experiencing language barriers during their care, which we postulate affected their overall satisfaction with the care received. Our findings suggest strategies to improve access to language-concordant care in this population are needed in Sri Lanka. Future research should focus on barriers to accessing oncology care among patients of different language proficiencies and predictors of patient satisfaction.
